# The women made it work: fuzzy transitive closure of the results chain in a dengue prevention trial in Mexico

**DOI:** 10.1186/s12889-017-4301-0

**Published:** 2017-05-30

**Authors:** Neil Andersson, Mario Beauchamp, Elizabeth Nava-Aguilera, Sergio Paredes-Solís, Mateja Šajna

**Affiliations:** 1 Centro de Investigación de Enfermedades Tropicales (CIET), Universidad Autónoma de Guerrero, Acapulco, Guerrero Mexico; 2 Department of Family Medicine, McGill University, Montreal, Canada; 3 CIETcanada, 160 George Street, Ottawa, Canada; 4 Department of Mathematics and Statistics, University of Ottawa, Ottawa, Canada

**Keywords:** Dengue, Community mobilisation, Behaviour change model, Fuzzy transitive closure

## Abstract

**Background:**

A modified theory of planned behaviour (acronym CASCADA) proposes that **C**onscious knowledge precedes a change in **A**ttitude, which in turn precedes positive deviations from negative **S**ubjective norms, intention to **C**hange, perception of **A**gency to change, **D**iscussion of possible action, and **A**ction itself. We used this as a results chain to investigate gender-specific behaviour dynamics in chemical-free dengue prevention.

**Methods:**

Secondary analysis of the Mexican arm of a cluster randomised controlled trial used household survey data on intermediate outcomes of dengue prevention behaviour. We used a matrix of odds ratios between outcomes, transformed to a symmetrical range (−1, 1), to compute fuzzy transitive closure of the results chain for control and intervention clusters, then for male and female respondents separately in each group. Transitive closure of a map computes the influence of each factor on each other factor, taking account of all influences in the system. Cumulative net influence was the sum of influences across the results chain.

**Results:**

Responses of 5042 women and 1143 men in 45 intervention clusters contrasted with those of 5025 women and 1179 men in 45 control clusters. Control clusters showed a distal block (negative influence) in the results chain with a cumulative net influence of 0.88; intervention clusters showed no such block and a cumulative net influence of 1.92. Female control respondents, like the overall control picture, showed a distal block, whereas female intervention responses showed no such blocks (cumulative net influence 0.78 and 1.73 respectively). Male control respondents showed weak distal blocks. Male intervention responses showed several new negative influences and a reduction of cumulative net influence (1.38 in control and 1.11 in intervention clusters).

**Conclusions:**

The overall influence of the intervention across the results chain fits with the trial findings, but is different for women and men. Among women, the intervention overcame blocks and increased the cumulative net influence of knowledge on action. Among men, the intervention did not reinforce prevention behaviour. This might be related to emphasis, during the intervention, on women’s participation and empowerment. The fuzzy transitive closure of the CASCADA map usefully highlights the differences between gender-specific results chains.

**Trial registration:**

ISRCTN27581154.

## Background

A recent cluster randomised controlled trial in Mexico and Nicaragua showed a convincing impact of evidence-based community mobilisation on risk of dengue [1]. The *Camino Verde* intervention had a positive effect on all outcome parameters (serological evidence of dengue virus infection in children, self-reported dengue cases and all conventional vector indices). Earlier trials of community-based interventions have shown impact on entomological indices [2–9], but the *Camino Verde* trial was the first to show impact on dengue virus infection. It is likely that there will be increased emphasis on community-mobilisation for prevention of dengue and other diseases borne by the *Aedes aegypti* vector, especially with evidence from the *Camino Verde* trial and from other studies [10–13] of the lack of effectiveness of the use of the organophosphate pesticide temephos in household water stores in reducing vector indices and dengue infection [14].

While the *Camino Verde* intervention had impact, it did not eradicate the vector or dengue virus infection in the intervention clusters. It is important to know how interventions of informed community mobilisation for dengue prevention achieve an impact and how they might be fine-tuned to increase their impact. Complex interventions can change many aspects of human behaviour. In the impact study of the *Camino Verde* trial, we measured a number of intermediate behavioural outcomes. These are described in the CASCADA model, based on the theory of planned behaviour [15, 16]. This extends the knowledge, attitudes and practices (KAP) model of behaviour change popularised in the 1960s [17].

Recognising the well-documented limitations of the KAP model [18, 19], our acronym CASCADA [20, 21] describes a partial order of intermediate outcomes through **C**onscious knowledge, **A**ttitudes, **S**ubjective norms and positive deviation from these, intention to **C**hange, **A**gency (individual and collective) to make change, **D**iscussion of possible action and, finally, **A**ction or change of practice. We used the CASCADA model as the basis of a randomised trial of an intervention to increase vaccination uptake in Pakistan [22, 23] and in analysis of a cross-sectional study in southern Africa [24]. A qualitative analysis of narratives of young women in three southern African countries validated the CASCADA model in the case of action to prevent gender violence [25].

**Fig. 1:**
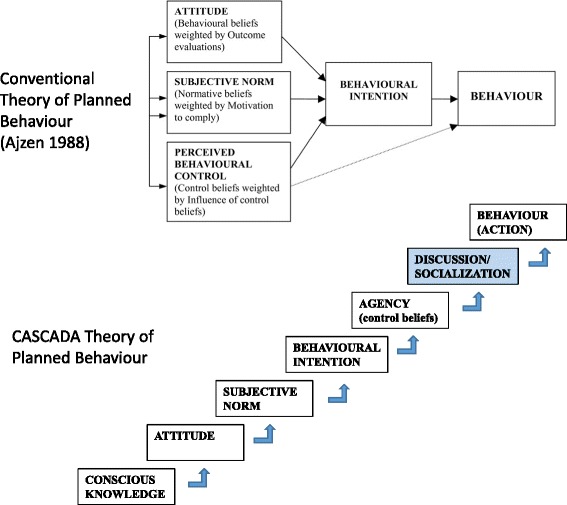
The CASCADA results chain compared with the conventional theory of planned behaviour

Our CASCADA model uses the constructs of the conventional theory of planned behaviour. Figure 1 shows the differences between CASCADA and the conventional theory of planned behaviour [15]. First, we propose CASCADA as a partial order, not an invariable or linear sequence, but different to the parallel constructs seen in the conventional theory: knowledge generally precedes attitude; attitude shifts generally precede a positive deviation from a negative subjective norm; and so on. Second, the CASCADA model adds “Discussion/Socialisation” as a measurable construct between Agency (perceived behaviour control) and Action (behaviour). We base this on the observation that where behaviour change is an essentially SOCIAL outcome, it is reasonable to expect discussion or socialisation one step before implementation.

The *Camino Verde* trial reported unremarkable differences in CASCADA outcomes between intervention and control clusters: there was a significant difference in only one intermediate outcome, Agency or collective self-efficacy (RRR 9.6% 95%CI 3.4–15.8) [1].

Here we report a secondary analysis of the impact of the *Camino Verde* intervention on the outcomes in the CASCADA results chain in all respondents and in men and women separately, in the Mexican arm of the trial, as part of our efforts to understand the gender-specific individual responses to the community-led intervention.

## Methods

The methods and results of the *Camino Verde* trial are described in detail elsewhere [1]. This secondary analysis used data from the impact survey of the trial in Mexico in 2013. Fieldworkers interviewed household respondents in 45 intervention and 45 control clusters in the coastal region of Guerrero state. The *Camino Verde* CASCADA model included the following variables: **C**onscious knowledge (C) was the ability to identify a physical sample of a mosquito larva (the interviewer showed a physical larva and took mention of “mosquito” in the response to indicate conscious knowledge of the mosquito life cycle). **A**ttitude (At) to vector control was the response to the question: Do you consider it worthwhile (*vale la pena*) to spend time and money every week to eliminate mosquito breeding sites (*criaderos*) in your house. **S**ubjective norm (S) was derived from the question: Do you think your neighbours consider it worthwhile to spend time …. Intention to **C**hange (iC) used the question: Do you plan to spend time and money each week to eliminate mosquito breeding sites in your house. **A**gency (Ag) derived from answers like “myself” or “ourselves” to the question: Who is responsible for control of mosquito breeding sites. **D**iscussion (D) about prevention derived from the response to the question: How often do you chat with your neighbours about how to control mosquitos. These two constructs, Agency and Discussion, replace perceived behaviour control in a more usual theory of planned behaviour [26]: Agency refers to both self-efficacy and collective efficacy; we included Discussion as a linkage to factors that facilitate the performance of the behaviour. **A**ction (A) focused on participation in collective activities in the neighbourhood to control mosquito breeding sites.

### Analysis

The analysis involved computation of fuzzy transitive closure technique of each CASCADA results chain. We used the command TABMAT in CIETmap [27] -- open source software with an interface in the statistical programming language R -- to generate a matrix of unadjusted odds ratios between each pair of variables in the CASCADA partial order. Using the formula

$$ 1\hbox{-} \left(2/\left(\mathrm{OR}+1\right)\right) $$

we converted the odds ratios to the interval [−1,1] and inputted the resulting matrix to FuzzyTC [28], a software package that computes the fuzzy transitive closure of a cognitive map [29].

Transitive closure has been used for analysis of cognitive maps in public health [30] and medicine [31]. Probabilistic transitive closure is applicable to fuzzy cognitive maps where the weights for the arcs can be interpreted as probabilities of the corresponding causal relations [28, 29]. For the CASCADA results chain, however, the fuzzy transitive closure model is more suitable [28].

This model assumes that the weight *w*(*A;B*) of the arc (*A;B*) is the *strength* of the direct influence of factor *A* on factor *B.* It is positive if an increase in *A* results in an increase in *B or* if a decrease in *A* results in a decrease in *B*. It is negative if an increase in *A* results in a decrease in *B*, or decrease in *A* results in an increase of B. For transitive closure to be meaningful, these influences must be *direct* and not involve any other factors under consideration. Also, the weights must be in the interval [−1,1], which is fulfilled after the transformation of the odds ratios into this range.

An arc (A;B) in the transitive closure arises either from a direct influence (arc) (A;B) in the CASCADA map, or else from a *walk* (sequence of arcs) of direct influences starting at A and ending at B. The weight of the arc (A;B) in the fuzzy transitive closure is the maximum weight of any of the walks from A to B, and the weight of a walk is the minimum of the weights of any of its arcs. Thus, the arcs of the CASCADA map can be seen as links in a chain. An arc in the fuzzy transitive closure is considered as strong as the bundle of chains that links its ends, and the strength of a chain is measured by the strength of its weakest link.

The resulting weight of the arc (A;B) in the transitive closure is interpreted as the overall (positive or negative) influence of factor A on factor B, which takes into account all direct influences in the original map.

FuzzyTC returns the fuzzy transitive closure in the form of two matrices, one of positive and the other of negative-weight arcs. We added these two matrices to obtain the *net fuzzy transitive closure.* Finally, we calculated the *cumulative net influence* (in the range − 6 to 6) as the sum of the weights of the influences in the fuzzy transitive closure over all arcs of the original CASCADA chain.

## Results

**Fig. 2:**
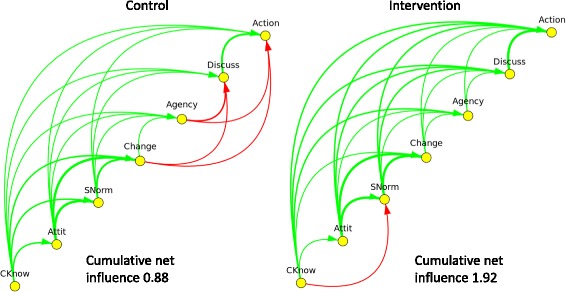
Net fuzzy transitive closure of the CASCADA results chain of responses in control and intervention clusters in the *Camino Verde* trial

We analysed responses of 6185 people (5042 women and 1143 men) in the intervention group and 6204 (5025 women and 1179 men) in the control group. The left side of Fig. 2 shows the net transitive closure of the CASCADA map derived from responses in the 45 control communities. Green arcs represent the positive net influences and red arcs the negative net influences, all in the direction of Action. Thicker lines indicate stronger influences. Conscious knowledge has an influence on Attitude, Subjective norm, and each subsequent step in the CASCADA. Attitude influences Subjective norm, Change intention, and each subsequent step in the CASCADA. In the control clusters, there is a negative influence of intention to Change on Discussion and on Action; also of Agency on Discussion; and of Agency on Action. The cumulative net influence across the CASCADA sequence is 0.88 out of a possible maximum value of 6 (which would be attained if every outcome in the CASCADA results chain had the strongest possible positive influence on the next factor in the chain).

The right side of Fig. 2 shows the analysis of the net transitive closure of the CASCADA map of outcomes in the 45 intervention clusters. The negative influences in the control clusters – red arrows between intention to Change, Agency and Action – do not appear in the intervention responses; instead there are strongly positive influences in the intervention group. The net influence of these variables on Action is consequently much higher in the intervention than in the control clusters (1.92 versus 0.88). The only negative arc is between Conscious knowledge and Subjective norms, the implication being that the more people in intervention sites knew about dengue prevention, the less they recognised incompatible norms of their neighbours.

**Fig. 3:**
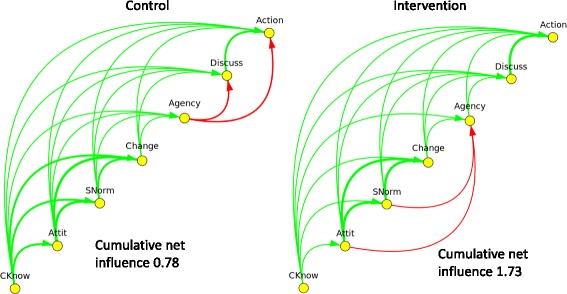
Net fuzzy transitive closure of the CASCADA results chain of female responses in control and intervention clusters in the *Camino Verde* trial

Among female respondents (Fig. 3), the control group showed much the same picture as did the overall control group. The intervention group, again like the overall picture, showed positive arcs replacing the negative arcs from Agency to Discussion and from Agency to Action. Two negative arcs emerged: between Attitudes to prevention and Agency, and Subjective norms to Agency. The cumulative net influence increased from 0.78 to 1.73 with the intervention.

**Fig. 4:**
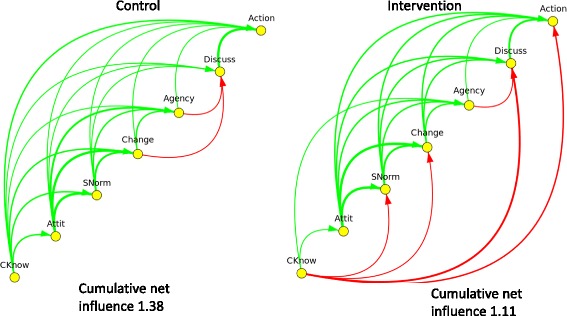
Net fuzzy transitive closure of the CASCADA results chain of male responses in control and intervention clusters in the *Camino Verde* trial

Figure 4 shows the findings for male respondents. Male control responses showed similar patterns to female control responses but male intervention responses were very different to female intervention responses. Four strongly negative arcs emerged among men in intervention clusters, with Conscious knowledge negatively related to Subjective norms, intention to Change, Discussion and Action. Among male respondents, the cumulative net influence *decreased* from 1.38 in the control clusters to 1.11 in the intervention clusters.

## Discussion and conclusions

Fuzzy transitive closure allowed scrutiny of the proposed results chain underlying dengue prevention action in the *Camino Verde* trial. The cumulative net influence across the CASCADA partial order of intermediate outcomes is compatible with the main finding of the trial, illustrating a likely mechanism of the positive influence on participant behaviour. Positive arcs between Change intention or Agency and Discussion or Action replaced negative arcs in the control clusters, probably related to the multiple opportunities to discuss prevention offered by the intervention, and reflected in the increase in collective efficacy (Agency) reported in the original study [1].

The transitive closure of the CASCADA model emerging from male and female responses, however, is quite different. Among women, the intervention appears to have overcome blocks in the distal partial order and increased the net influence of Conscious knowledge on Action. It seems plausible that this accounts for most of the measured impact in the trial.

Responses from men suggest the *Camino Verde* intervention did not work well for them, in the important sense that it did not generate or reinforce positive prevention behaviour in this group. If anything, the intervention seems to have weakened the existing influence of knowledge on action shown in the control group, quite possibly demotivating men.

It is possible that the male responses included in our analysis represent a selected and minority view – there were many fewer male than female household respondents, and the male responses came from men who were at home at the time of the survey, when most men were out at work. The findings may also be related to the intentional emphasis of the intervention on women’s participation and empowerment. The *Camino Verde* intervention was gendered in the sense that it engaged, mobilised and empowered women. The negative reaction of men might have represented some sort of push-back in the context of women engaging in community issues that might ordinarily have been a male domain. This phenomenon is well recognised in other spheres of development involving women’s empowerment [32–34]. In a broader and probably more correct sense of gendering interventions, we should also have considered and addressed the potential effect of women’s engagement on their spouses.

Fuzzy transitive closure operationalises the CASCADA partial order by quantifying the sequential relationship (for example, Knowledge leading to Attitudes) in the context of all other relationships between Knowledge and Attitudes, in both directions. The resulting quantification is context and subgroup specific, as the relative influence of the network of different constructs will change with context. Fuzzy transitive closure incorporates the influences of all other arcs, to generate the summary that is very different for men and women even in the same communities. The difference is informative, fuelling discussions about intervention gender dynamics.

As far as we can ascertain, this is the first use of transitive closure to analyse gender specific results chains in behaviour change interventions. By treating the intermediate outcomes as a network with each node potentially related to every other node, transitive closure provides a coherent summary of the overall relation between knowledge and preventive action. It allows us to evaluate the results chain in a way that appropriately emphasises the weakest link in the chain. It also illustrates in a compelling way where “blocks” in the CASCADA need to be confronted and, in the counterfactual comparison allowed by the *Camino Verde* trial, whether the intervention overcomes these blocks.

Our conclusion is that future dengue prevention interventions have to work effectively with both women and men, perhaps beginning with gender-stratified action planning and then bringing together the two groups for strategic discussions about who tackles what part of the problem.
